# Increased Seroprevalence of Typhus Group Rickettsiosis, Galveston County, Texas, USA

**DOI:** 10.3201/eid2901.221206

**Published:** 2023-01

**Authors:** Lucas S. Blanton, Maria A. Caravedo Martinez, Nicole Mendell, Alejandro Villasante-Tezanos, David H. Walker, Donald Bouyer

**Affiliations:** University of Texas Medical Branch, Galveston, Texas, USA

**Keywords:** Rickettsia, bacteria, vector-borne infections, parasites, zoonoses, Rickettsia typhi, murine typhus, endemic typhus, flea-borne typhus, typhus group rickettsiosis

## Abstract

Whether increases in typhus group rickettsiosis in Galveston County, Texas, USA, are caused by increased recognition or true reemergence is unclear. We conducted a serosurvey that demonstrated *Rickettsia typhi* antibodies increased from 1.2% in 2013 to 7.8% in 2021 (p<0.001). These findings support pathogen reemergence rather than enhanced recognition alone.

Murine typhus is an acute febrile illness caused by fleaborne *Rickettsia typhi* bacteria (*1*). Associated with rats and their fleas (*Xenopsylla cheopis*) throughout much of the world, contemporary murine typhus cases in the United States are thought to be related to a zoonotic cycle involving Virginia opossums (*Didelphis virginiana*) and cat fleas (*Ctenocephalides felis*) (*2*). Although murine typhus was once quite prevalent in the United States, vector control campaigns led to a drastic decrease in disease incidence (*3*), limiting endemic foci to parts of southern California and the most southern counties of Texas (*1*,*2*). We previously reported the reemergence of typhus group rickettsiosis (TGR), likely murine typhus, in Galveston, Texas, USA, after decades of apparent absence (*4*). Since then, incidence of reported cases has increased in Galveston County and throughout Texas (*5*,*6*). Whether the recent increase in TGR represents reemergence due to regional changes in ecologic factors or newfound physician awareness is unclear.

Although increased clinical recognition has undoubtedly played a role in diagnosing cases, we believe that the disease has reemerged through a change in regional reservoir and vector dynamics. The increased prevalence and northward distribution of TGR in Texas contributes to our belief (*6*). When investigating the reemergence of TGR in Galveston in 2013, we conducted a serosurvey of Galveston residents, 1.2% of whom demonstrated *R. typhi* seropositivity (*4*). In this study, we aimed to determine if seroprevalence has remained static, suggesting clinical recognition as the sole driver behind an increase in reported cases, or if seroprevalence has increased, suggesting a true increase in *R. typhi* exposure.

We assessed the prevalence of *R. typhi* reactive antibodies by repurposing serum samples collected from 528 residents of Galveston County. The samples were scheduled to be discarded after routine clinical testing from outpatient clinics during the winter of 2021. We used electronic medical records to extract the age, sex, and postal code of residence associated with each serum sample and included specimens with postal codes from Galveston County communities in the study (Table). We excluded duplicate specimens and specimens from postal codes outside Galveston County. We screened serum samples for *R. typhi* IgG by using indirect immunofluorescence assay (IFA) at a titer of 1:128, as described in our prior serosurvey (*4*). We used Alexa Fluor 488-conjugated AffiniPure goat anti-human IgG (Fc_γ_ fragment specific) (Jackson Immuno Research Laboratories, https://www.jacksonimmuno.com) as a conjugate antibody at a dilution of 1:800. We used convalescent serum from a patient previously diagnosed with TGR at a titer of 1:1,000 as a positive control. We used a 1:128 titer of serum from a healthy seronegative donor as a negative control. We established endpoint titers for reactive specimens. We performed Western blot analysis by using *R. typhi* as the antigen to confirm specificity of IFA-reactive specimens to rickettsial outer membrane protein B, as described elsewhere (*4*,*7*). We used χ^2^ test to compare categorical data on reactive versus nonreactive specimens with data from 2013 and compared geometric mean titers with data from 2013 by using Mann-Whitney U test on log transformed titers. We performed all statistical analyses in SAS version 9.4 (SAS Institute, Inc., https://www.sas.com). The study was approved by the University of Texas Medical Branch institutional review board (protocol no. 20-0259).

**Table:** Serum samples reactive against *Rickettsia typhi* in a study of seroprevalence of typhus group rickettsiosis, Galveston County, Texas, USA*

Community	No. tested	No. (%) reactive
Galveston County, total	528	41 (7.8)
City of Galveston	146	12 (8.2)
League City	100	12 (12.0)
Texas City	90	5 (5.6)
La Marque	33	4 (12.1)
Dickinson	62	4 (6.5)
Hitchcock	20	1 (5.0)
Kemah	6	1 (16.7)
Port Bolivar	8	1 (12.5)
Santa Fe	38	1 (2.6)
Bacliff	14	0
Friendswood	11	0

*Reactive was defined as samples positive by immunofluorescence assay at a reciprocal titer >128 and a confirmatory Western blot test.

We tested 528 serum samples from persons across Galveston County (Table). Most (376/528, 71.2%) specimens were from female patients with a median age of 51 years. IFA reactivity was demonstrated in 46 (8.7%) persons, among whom 41 (7.8%) had confirmed seroreactivity (i.e., reactive by both IFA and Western blot). The geometric mean reciprocal titer was 521, which is much higher than the value of 197 we noted in 2013 (p<0.04). Seropositive specimens were found in all communities except Friendswood and Bacliff (Figure). Compared with the 1.6% (8/500) seroprevalence in the city of Galveston in 2013, which we obtained with the same methodology used in this study, we noted a higher seroprevalence in both the city of Galveston (8.2%, 12/146; p<0.001) and throughout Galveston County (7.8%, 41/528; p<0.001) in 2021.

**Figure:**
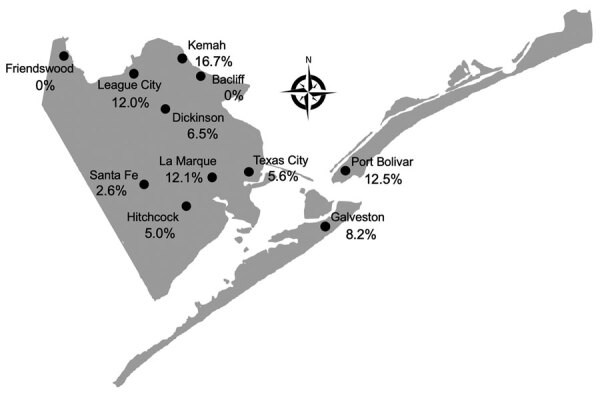
*Rickettsia typhi* seroprevalence in communities of Galveston County, Texas, USA, 2021. We tested 528 serum samples from persons across Galveston County by using indirect immunofluorescence assay and Western blot tests. Percentage seropositivity for each area is shown.

The increased seroprevalence of *R. typhi* reactive antibodies supports the hypothesis that the increase in TGR cases reported in Galveston County is because of pathogen reemergence in this region, rather than enhanced clinical recognition alone. Considering the kinetics of *R. typhi* antibody (*8*), the higher geometric mean titer supports more recent seroconversion in the 2021 sample cohort, further supporting our hypothesis. Regional changes in the zoonotic transmission cycle could be contributing to this increase. Such changes might include a shift from the classic rat–rat flea urban transmission cycle to one involving opossums and cat fleas. In addition, another study demonstrated high *R. typhi* seropositivity (66.7%) in opossums and a high proportion of *R. typhi*–infected fleas (7%) collected from these animals in Galveston (*9*). More studies are needed to understand the ecology of TGR and risk to public health. Clinicians and public health officials should be aware of the increase of *R. typhi* seropositivity in the Galveston area and recognize the signs and symptoms of murine typhus.
